# Anticipating the unimaginable: Heat Health Warning Systems in Europe and Australia

**DOI:** 10.1093/eurpub/ckac131.162

**Published:** 2022-10-25

**Authors:** M Parkes, A Bone, D Csutoros

**Affiliations:** 1 School of Public Health, LSHTM, London, UK; 2 Strategy, Policy & Outbreak Response, Covid-19 Public Health Division, Victoria Health, Melbourne, Australia; 3 Metro North Public Health Unit, Queensland Health, Brisbane, Australia; 4 Health Protection Branch, Victoria Health, Melbourne, Australia; 5 School of Medicine, University of Exeter, Exeter, UK; 6 School of Public Health and Preventive Medicine, Monash University, Melbourne, Australia; 7 Victoria Health, Melbourne, Australia

## Abstract

**Background:**

It has long been a maxim of climate and health research that heatwaves kill more people than all other natural disasters combined. Heatwave frequency, severity and duration have increased as climate change intensifies, and recorded temperatures during recent heat events now regularly surpass previous worst-case projections. Temperate zones, between 40°-60° north or south of the Equator, are particularly vulnerable, as small variations in mean temperature can trigger large-scale increases in morbidity, mortality and concomitant health service strain. Heat Health Warning Systems (HHWSs) combine temperature forecasts with public health actions to mitigate these impacts. However, in the absence of a consensus definition of heatwaves multiple systems have developed, and comparative analysis of the effectiveness of different HHWS and their interventions are hindered by lack of a common threshold metric for defining and predicting heatwave severity.

**Methods:**

This paper provides a comprehensive review of current HHWS and their evidence base in temperate zones in Europe, the United Kingdom and Australia - contiguous landmasses containing multiple jurisdictions with high heterogeneity in local HHWSs, in which single heatwave events trigger vastly different public health responses. A systematic review of available published and grey literature was undertaken to generate a schema of HHWSs in these zones. Results were then narrowed to review and synthesise evidence for each, with a focus on threshold effectiveness in predicting health impacts.

**Results and conclusions:**

Over twenty distinct HHWS are reviewed, with substantial variation in the evidence for their effectiveness. We make the case for a unified threshold metric for defining heatwaves, to facilitate research and identify warning systems which accurately predict health impacts and effectively communicate risk.

**Key messages:**

• Lack of definitional consensus and heterogeneity in threshold metrics hinders comparison of Heat Health Warning Systems.

• Accurate prediction and rapid communication of heat risk is crucial to prevent health impacts of heatwaves.

